# Structural characterization of glycinamide-RNase-transformylase T from *Mycobacterium tuberculosis*

**DOI:** 10.1080/22221751.2019.1707716

**Published:** 2020-01-02

**Authors:** Cong Chen, Zuliang Liu, Liguo Liu, Jianmin Wang, Qi Jin

**Affiliations:** a NHC Key Laboratory of Systems Biology of Pathogens, Institute of Pathogen Biology, and Center for Tuberculosis Research, Chinese Academy of Medical Sciences and Peking Union Medical College, Beijing, P. R. People’s Republic of China; b Collaborative Innovation Center for Diagnosis and Treatment of Infectious Diseases, Hangzhou, PR People’s Republic of China

**Keywords:** *Mycobacterium tuberculosis*, glycinamide-RNase-transformylase T, crystal structure, purine salvage pathway, anti-TB drug development

## Abstract

Enzymes from the purine salvage pathway in *Mycobacterium tuberculosis* (*Mtb*) have been regarded as an attractive target for the development of anti-bacterial drugs. Although this pathway has not been extensively studied in *Mtb*, it has been identified as essential for growth and survival. Glycinamide-RNase-transformylase T (PurT) is found only in some specific bacteria including *Mtb* and utilizes ATP-dependent ligation to catalyze the formylation of 5′-phosphoribosyl-glycinamide (GAR) in the third reaction of the de novo purine salvage pathway. In the study, we determined the crystal structure of *Mtb*PurT at a resolution of 2.79 Å. In contrast to *Pyrococcus horikoshii OT3* PurT (phBCCPPurT), *Mtb*PurT exhibits an “open” conformation, which results in a broader ATP-binding pocket and thus might facilitate the entry and exit of the cofactor. Additionally, active site superposition with *E.coli* PurT (*Ec*PurT) showed that residues involved in the ATP-binding site in *Mtb*PurT exhibited structural similarity but had notable difference in the GAR-binding site. The loop 383-389 in *Mtb*PurT was much shorter and shifted 5.7 Å away from the phosphate of the GAR substrate. The different GAR-binding mode might result in a large conformational change in *Mtb*PurT, and would provide a possible opportunity for anti-TB drug development.

## Introduction

Tuberculosis (TB) is considered a serious global health issue threatening humans worldwide, claiming approximately 2 million lives each year [1]. It is a chronic disease caused by a pathogenic bacterium named *Mycobacterium tuberculosis* (*Mtb*) and spreads from person to person through the air. TB generally infects the lungs but can also infect other parts of the body [2–4]. Current TB therapy involves a regimen of four vaccines for effective control in developed countries [5,6]. However, these drugs still have the limitations of high cost and prolonged administration period in developing countries. In particular, the prevalence of multidrug resistance continues to increase at an alarming rate, resulting in morbidity and mortality [5]. Therefore, exploring new drugs and vaccines against latent bacteria would provide a better insight into the resistance of *Mtb* strains and achieve effective control of TB in a short period, particularly in developing countries.

Nucleoside pathways can be a good source of metabolic energy and are considered a prospective target for new potential drug leads. The enzymes involved in this pathway usually have distinct characteristics from those of human counterparts. One of the attractive pathways for the development of nucleoside analogs with anti-TB activity is the purine salvage pathway [7,8]. Purine salvage enzymes are useful in the treatment of TB infection because of their capacity to permit the metabolism of nucleoside analogs to active compounds [9–11]. To date, several homologues to enzymes involved in the purine salvage pathway have been identified based on the genome sequence of *Mtb* H37Rv [12]. However, little is known about purine metabolism in *Mtb*; thus, we need to investigate the enzymes involved in the salvage of purine nucleosides. A comprehensive understanding of those enzymes would provide insight into the identification of nucleoside analogs and affect the *Mtb* strain.

**Figure 1.**
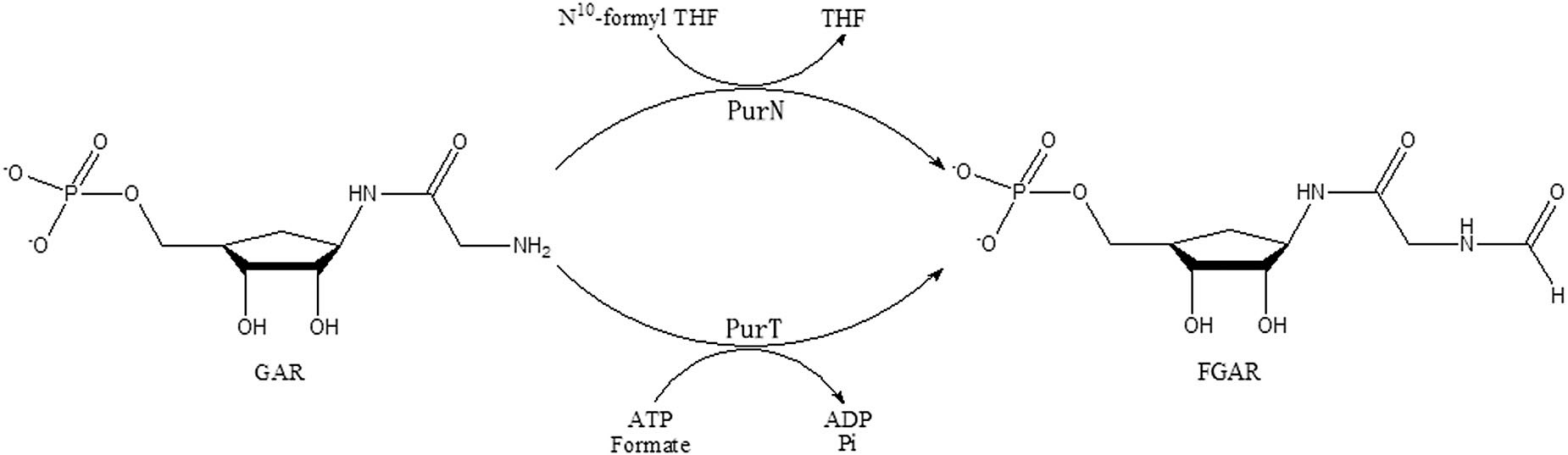
**Scheme of the reactions catalyzed by two GAR transformylases, PurT and PurN.** In the third step of the de novo purine nucleotide biosynthesis pathway, PurT uses formate and ATP to catalyze GAR to N-formyl-GAR (FGAR), while PurN uses 10-formyltetrahydrofolate in this reaction.

Total ten steps are involved in the purine salvage pathway to convert phosphoribosyl-pyrophosphate (PRPP) into inosine 5′-monophosphate (IMP) [13,14]. In the third step, two enzymes PurT (glycinamide-RNase-transformylase T) and PurN (glycinamide-RNase-transformylase N), catalyze the formylation of 5′-phosphoribosyl-glycinamide (GAR) to obtain formyl-phosphoribosyl-glycinamide (FGAR) using different formyl donors [11,15–17] (Figure 1). PurT utilized ATP-dependent ligation of formate as a formyl donor instead of the cofactor *N*^10^-formyltetrahydrofolate (N^10^-formylTHF) by PurN, which is found in both prokaryotes and eukaryotes [13,18]. In contrast, PurT is found only in some eubacteria and several thermophilic archaebacteria, including *Mtb* [19,20]. Therefore, PurT may represent a good anti-TB drug target. In this study, the three-dimensional structure of *Mtb*PurT was successfully solved. Structural studies on *Mtb*PurT would lead to a better understanding of the catalytic mechanism as well as deeper insight into novel drug development against *Mtb* strains to combat emerging multidrug resistance.

## Materials and methods

### Protein expression and purification

The open reading frame sequence encoding PurT (Rv03894) was amplified from the genomic DNA of *Mtb* H37Rv by polymerase chain reaction (PCR). The PCR amplified minigene (Gene ID: 886032, bases 1–1260 nt) was purified, digested with *EcoR*I and *Xho*I and cloned into the same restriction sites of pProExHta (Novagen, Madison, Wisconsin, USA). Clones were selected by PCR and restriction mapping. The plasmid was transformed into *E.coli* strain BL21 (DE3) for protein expression. The *E.coli* BL21 (DE3) transformant was cultured in 1000 ml of fresh LB medium (10 g of Bacto tryptone, 5 g of yeast extract and 10 g of NaCl per litre of solution) containing ampicillin (100 μg/ml) at 37°C. When OD_600_ ∼0.8, protein expression was induced by 0.0625 mM isopropyl-*β*-d-thiogalactoside (IPTG) at 37°C for 4 h. The cells were harvested by centrifugation at 6,520 g for 1 h at 4°C, washed with phosphate buffered saline (PBS) twice and then stored at −80°C.

The harvested cells were resuspended in lysis buffer (50 mM Tris-HCl, pH 7.5, 250 mM NaCl, 5 mM imidazole) and homogenized by sonication. The suspended lysate was centrifuged at 15,930 g for 1 h to remove cell debris. The clear supernatant was filtered (pore diameter 0.45 µm; Sartorius, Goettingen, Germany) and applied to a column of nickel-NTA beads (Qiagen, Hilden, Germany) pre-equilibrated with lysis buffer. The column was loaded with 70 column volumes of slurry and then washed with 15 column volumes of washing buffer (50 mM Tris-HCl pH 7.5, 250 mM NaCl, 30 mM imidazole), then eluted with 5 column volumes of elution buffer (50 mM Tris-HCl pH 7.5, 10 mM NaCl, 300 mM imidazole). Fractions containing *Mtb*PurT were pooled and concentrated in 50 mM Tris, pH 8.0 by ultrafiltration using a Centriprep YM-10 (Millipore Corporation, Bedford, MA, USA). The *Mtb*PurT was further purified by ion-exchange chromatography on an HQ20 column (Perseptive Biosystems, Foster City, CA, USA) pre-equilibrated with 50 mM Tris buffer, pH 8.0. The protein was eluted at ∼0.35 M NaCl and concentrated by ultrafiltration (Centriprep YM-10, Millipore Corporation, Bedford, MA, USA). *Mtb*PurT was finally purified by gel-filtration chromatography with a Superdex 200 10/300 GL column (GE Healthcare, Piscataway, NJ, USA) in gel-filtration buffer (50 mM Tris-HCl pH 8.0,100 mM NaCl). The fractions containing *Mtb*PurT protein were collected, exchanged into HEPES buffer (10 mM HEPES, pH 7.5) by ultrafiltration (Centriprep YM-10), and subsequently snap-frozen in liquid nitrogen at a −80°C.

*Pyrococcus horikoshii OT3* phosphoribosylglycinamide formyl transferase (*phBCCP*PurT, Gene ID: 1444201, bases 1–1293 nt) was custom synthesized and cloned into the pET28a vector (Novagen). The pET28a-*phBCCP*PurT construct was further confirmed by sequencing and then transformed into *E.coli* strain BL21 (DE3) for protein expression and purification as above.

## Crystallization and data collection

Purified *Mtb*PurT was concentrated to 8 mg/ml in 10 mM HEPES pH 7.5, and then screened for crystallization. Initial crystallization was carried out with Index Screen, PEGRx Screen and Crystal Screens I and II from Hampon Research (California, USA), Wizard Screens I, II, Cryo I and Cryo II from Emerald Biostrucures (Bainbridage Island, Washington, USA), JBScreen Basic HTS I, II from Jena Bioscience (Jena, Germany), Classics Suite I, II, JCSG Core I, II, III, IV, PHClear Suite I and II from Qiagen (Hilden, Germany) using the microbatch crystallization method at 18°C. After optimal crystallization, rod-shaped crystals were successfully obtained by the hanging drop vapour diffusion method. The crystallization experiments were conducted at 18°C, and the reservoir solution contained 0.06 M sodium acetate, pH 4.6, 6.0% PEG4000, 32% glycerol. For data collection the crystals were equilibrated in the reservoir solution adding 10% ethylene glycol to the well solution, flash cooled and stored in liquid nitrogen. Diffraction data were collected at 100 K by a Dectris Eiger X 16M detector at Shanghai Synchrotron Radiation Facility (SSRF) beamline BL17U1. All diffraction images were indexed, integrated and scaled using the programme XDS package [21].

### Structural determination and refinement

Structure of *Mtb*PurT was determined by the molecular replacement methods with the programme PHASER in CCP4i [22], and structure with PDB code 1EYZ (42% identity) was used as a search model. An initial model of *Mtb*PurT was manually built using Coot (Crystallographic Object-Oriented Toolkit) [23], and structural refinement was performed using the PHENIX programme [24]. The quality of the model was validated periodically during the model building/refinement process with the MolProbity programme [25]. The structural figures were prepared by the PyMOL [26]. All structural refinement statistics are listed in Table 1.

**Table 1.** Data collection and refinement statistics for *Mtb*PurT.

PDB ID	6KHR
Space Group	P3_1_21
Unit Cell (Å°)	A = 103.13 B = 103.13 C = 148.69 90 90 120
Number of molecules in ASU	2
Wavelength (Å)	0.9791
Resolution (Å)	45∼2.79 (2.94∼2.79)
*R*_merge_ (%)	26.6 (87.4)
*R*_pim_ (%)	9.8 (32)
I/sigma	8.0 (4.7)
Completeness (%)	99.4 (97)
Number of measured reflections	206579 (27385)
Number of unique reflections	23300 (3251)
Redundancy	8.9 (8.4)
Wilson B factor (Å^2^)	50.7
*R*_work_ /*R*_free_	0.217/0.242
Number of atoms	
Protein main chain	2676
Protein side chain	2210
Protein all atoms	4886
Water molecules	34
Other entities	0
All atoms	4920
Average B value (Å^2^)	
Protein main chain	60.5
Protein side chain	67.7
Protein all atoms	63.7
Water molecules	62.2
Other entities	0
All atoms	63.7
Rms deviations from ideal values	
Bonds (Å)	0.012
Angle (°)	1.316
Ramachandran plot statistics (%)	
Most favourable	90.0
Additionally allowed	9.1
Generously allowed	0
Disallowed	0

Values in parentheses are for the highest resolution shell. *R_merge_* = Σ_h_Σ_i_|*I_h,i_*-*I_h_*|/Σ_h_Σ_i_*I_h,i_*, where *I_h_* is the mean intensity of the *i* observations of symmetry related reflections of *h*. *R* = Σ|*F_obs_*-*F_calc_*|/Σ*F_obs_*, where *F_calc_* is the calculated protein structure factor from the atomic model (R_free_ was calculated with 5% of the reflections selected).

### Isothermal titration calorimetry assay

The isothermal titration calorimetry (ITC) assay was performed on a NANO ITC 2G at 37°C. Briefly, the proteins (*phBCCP*PurT, *Mtb*PurT and its mutations) were dissolved in PBS buffer and injected into the sample cell at the concentration of 100 μM, and then titrated with aliquots of 2 mM ATPγS or CTP solution. Data analysis were performed by NanoAnalyze software package (TA Instruments, New Castle, USA) using Independent Model.

### Site-directed mutagenesis

Primers for the *Mtb*PurT mutation (Table 2) were designed and commercially synthesized. *Mtb*PurT mutants were amplified by site-directed mutagenesis (KOD plus) according to the manufacturer’s protocol. The sequences of the mutants were confirmed through DNA sequencing. The expression and purification of mutants were performed as described above for native *Mtb*PurT.

**Table 2.** The primers used for amplification of *Mtb*PurT mutants.

Mutant	Position	Primer	
R131A	CGG112GCT	Forward	5′-GTTCGGCCGAACCCAGCGCTGAGCTGGCGA-3′
		Reverse	5′-AGCGCTGGGTTCGGCCGAACCCAGCAACA-3′
E217A	GAG648GCG	Forward	5′-CCTCGGGTGTGCGCCGCGTCGGTGGTCGA-3′
		Reverse	5′-GCGGCGCACACCCGAGGGCTCACTCC-3′
S218A	T649G	Forward	5′-TCGGGTGTGCGCCGAGGCGGTGGTCGAG-3′
		Reverse	5′-CCTCGGCGCACACCCGAGGGCTCACT-3′
V220A	TC656CG	Forward	5′-GCGCCGAGTCGGTGGCGGAGATCGAGT-3′
		Reverse	5′-CGCCACCGACTCGGCGCACACCCGAGG-3′

### Size-exclusion chromatography

A Superdex 200 10/300 GL column (GE healthcare) was equilibrated with PBS buffer and pre-calibrated using gel-filtration standards (thyroglobulin 670 kDa, γ-globulin 158 kDa, ovalbumin 44 kDa, myoglobin 17 kDa and vitamin B12 1.35 kDa). The *Mtb*PurT protein was loaded onto the column and eluted at 14.06 ml.

### Sequence and structure alignment

Sequence alignment was performed by MUSCLE software [27,28], and was illustrated and generated through a website ESPript 3.0 [29]. The structural alignment was performed by DALI Server [30]. The *Mtb*PurT/ATP/GAR model was generated based on the superposition of the *Ec*PurT/ATP/GAR (PDB code: 1KJ8) by the PyMOL, and the root-means square values (r.m.s.d) was 1.01 Å.

### Protein data bank accession number

Coordinates and structure factors of *Mtb*PurT have been deposited in the Protein Data Bank [31] with the accession number 6KHR.

## Results

### Structure determination of MtbPurT

The *Mtb*PurT crystal diffracted at 2.79 Å resolution at the SSRF and belonged to a space group P3_1_21. Its 3D-structure was determined by molecular replacement and refined to a R_work_/R_free_ of 0.217/0.242. The structure of *Mtb*PurT agrees well with the crystallographic data and expected geometric values. In the Ramachandran plot, 90.0% of the residues were in the most favourable region, and all of the residues were in the allowed region. There were two *Mtb*PurT molecules in the asymmetric unit. Data collection and refinement statistics are summarized in Table 1. Additional attempts to crystallize complex *Mtb*PurT/ATP or *Mtb*PurT/ADP were also made, unfortunately, no crystals grew.

### Overall architecture of MtbPurT

**Figure 2.**
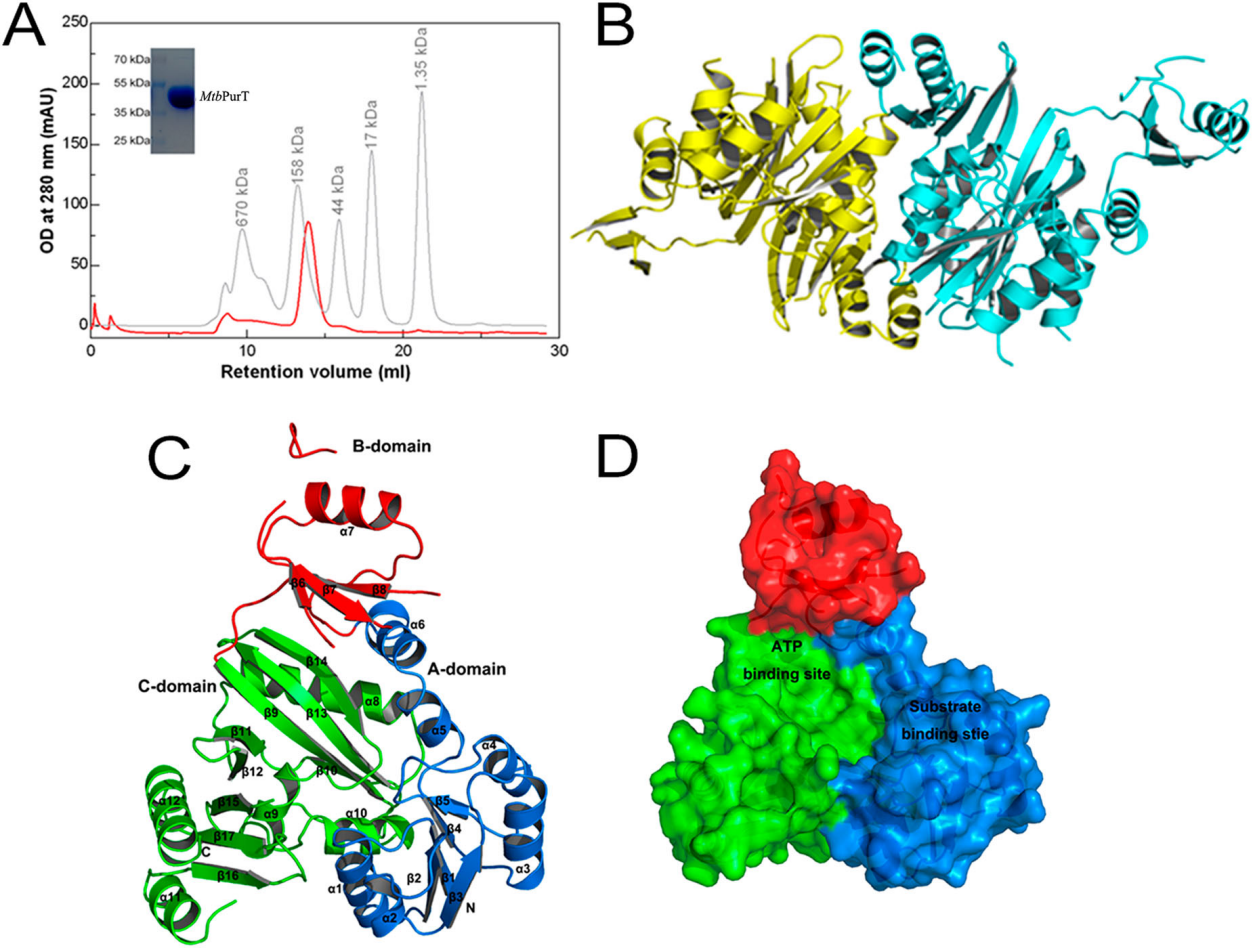
**Overall structure of *Mtb*PurT.** (A) Analytical gel-filtration chromatography of final purified *Mtb*PurT. A single peak was observed with molecular weight corresponding to the size of a dimer. The SDS-PAGE analysis of the final purified *Mtb*PurT was shown as upper insert. (B) Ribbon representation of the *Mtb*PurT dimer. The monomers are coloured yellow and cyan. (C) Subdomain structure of *Mtb*PurT. *Mtb*PurT structure comprises three subdomains, an A-domain at the N-terminus (coloured blue and labeled N), a B-domain in the middle (coloured red), and a C-domain at the C-terminus (coloured green and labeled C). The secondary structure elements are also labeled. (D) Surface representation of *Mtb*PurT. The possible ATP and substrate binding sites are marked.

The molecular weight of the *Mtb*PurT protein was estimated to be ∼90 kDa from gel-filtration chromatography, and the monomer protein was about 44 kDa from SDS-PAGE (Figure 2(A)). Therefore, the *Mtb*PurT protein exists as a dimer in solution. The final structure of *Mtb*PurT is well ordered, and electron densities for all residues were clearly interpretable, except in four parts (residues 111–113, 175–180, 189–198 and 205–215). The overall architecture of *Mtb*PurT forms a tight dimer in the asymmetric unit and the two monomers show a structure similarity to each other (Figure 2(B)). According to the *Mtb*PurT structure, the monomer of *Mtb*PurT consists of a small lid domain and a large *α*/*β* domain (Figure 2(C)). The small lid domain comprises one *α* helix (*α*7) and three *β* strands (*β*6, *β*7 and *β*8). The large domain mainly contains two *α*/*β* domains, *α* (1–6)/*β* (1–5) and *α* (8–12) and *β* (9–17). Each subunit can be divided into three domains, representing the A-, B- and C-domains. The A-domain contains 116 residues to form five parallel *β*-strands (1–5) surrounded by six *α*-helices (1–6) at the N terminus. The B-domain with 69 residues forms three antiparallel *β*-strands (6–8), and a helix covers the *β*-sheets. The C-domain consists of 189 residues and forms eight antiparallel *β*-strands (9–17) in the central and five *α*-helices (8–12) around. The B-domain is linked to both the A- and C-domains by a flexible loop with 6-residue and 4-residue, respectively. As shown from Figure 2(D), the A- and C-domains assemble together to form a core structure and provide a large central pocket for substrate binding. The B-domain forms a flexible lid and covers the active site for ATP binding.

### Comparison with apo-phBCCPPurT

**Figure 3.**
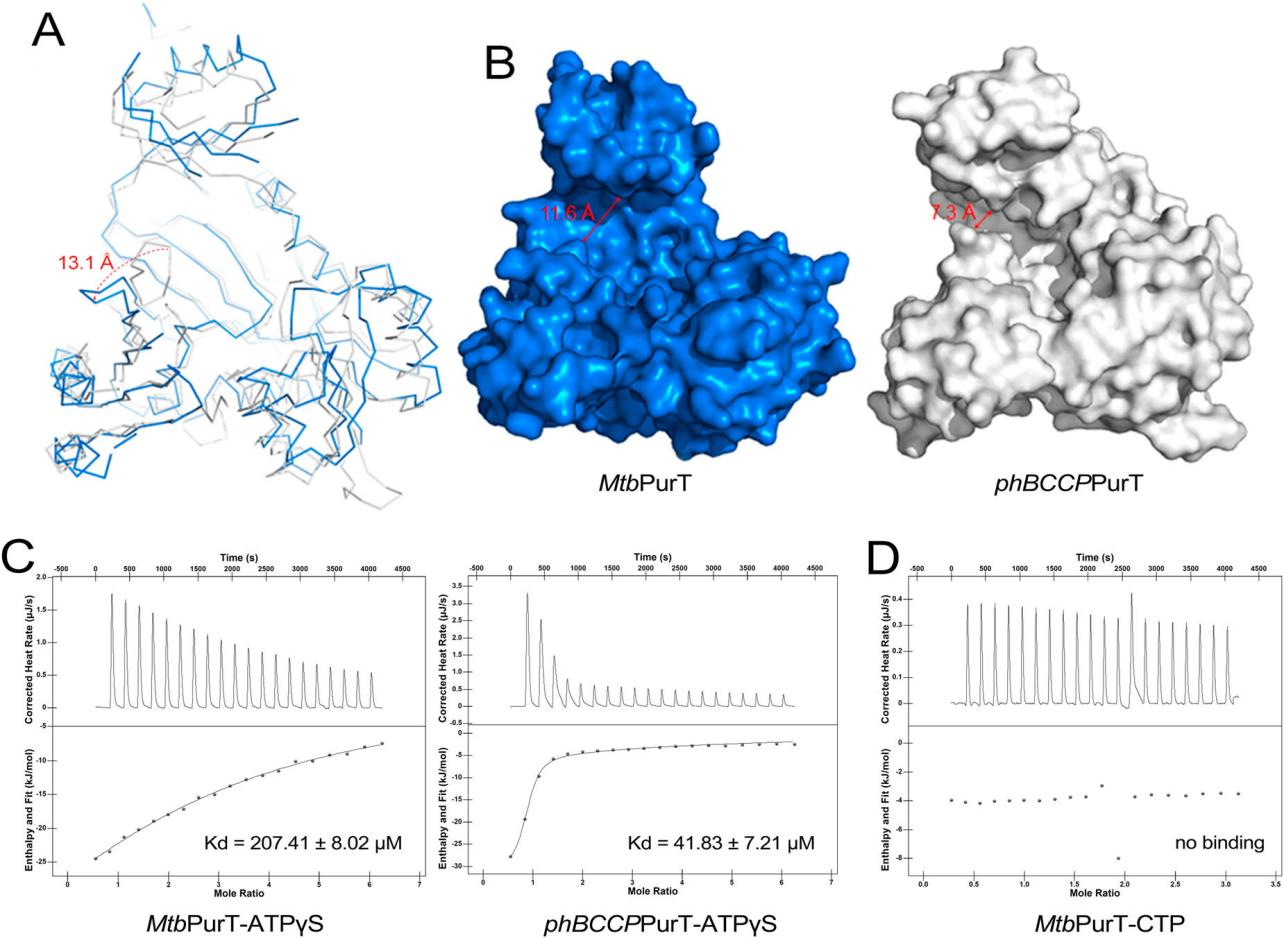
**Structural comparison of *Mtb*PurT with apo-*phBCCP*PurT.** (A) Superposition of *Mtb*PurT with apo-*phBCCP*PurT (PDB code: 2CZG, r.m.s.d: 1.30 Å). The shift of the loop in *Mtb*PurT from the positions in *phBCCP*PurT is marked as a dashed arrow in red. (B) Surface representation of the molecular surfaces in *Mtb*PurT and *phBCCP*PurT. (C) Isothermal titration calorimetry assay (ITC) demonstrates the ATP-binding affinity for *Mtb*PurT and *phBCCP*PurT. The calculated K*_d_* values are shown. (D) No binding affinity with CTP for *Mtb*PurT was detected by ITC.

To date, the structures of the apo form of *Pyrococcus horikoshii OT3* (*phBCCP*) and its complex with ADP and the structures of *E.coli* PurT (*Ec*PurT) complexed with ADP, ATP, ATP/GAR, AMPPCP and ATPγS complex have been successfully solved [19,32]. Figure 3(A) shows the structural superposition of the subunits of apo-*Mtb*PurT and apo-*phBCCP*PurT (PDB code: 2CZG, r.m.s.d: 1.30 Å). In contrast to *phBCCP*PurT, loop 252–255 between *β*11 and *β*12 in *Mtb*PurT shifted by 13.1 Å away from the cofactor binding site. This structural rearrangement in apo-*Mtb*PurT represents an “open” conformation. However, the apo-*phBCCP*PurT exhibits a “close” conformation. As a result, the pocket of *Mtb*PurT for the cofactor binding has a much broader opening with a width of 11.6 Å (distance between Ser182 and Asp251) than 7.3 Å (distance between Ser172 and Asp239) for *phBCCP*PurT (Figure 3(B)). As PurT may cause ATP hydrolysis (Figure 1), we utilized a non-hydrolysable ATP analog (ATPγS) to determine the ATP-binding affinity for *Mtb*PurT in this study. The isothermal titration calorimetry (ITC) assay was performed. As shown in Figure 3(C), *phBCCP*PurT binds ATPγS with an apparent dissociation constant (K_d_) of 41.83 ± 7.21 μM. And *Mtb*PurT recognizes ATPγS with a relatively low binding affinity, with the K_d_ value of 207.41 ± 8.02 μM. No binding affinity with CTP was detected for *Mtb*PurT (Figure 3(D)).

### Structural similarity with the ATP-grasp fold family

**Figure 4.**
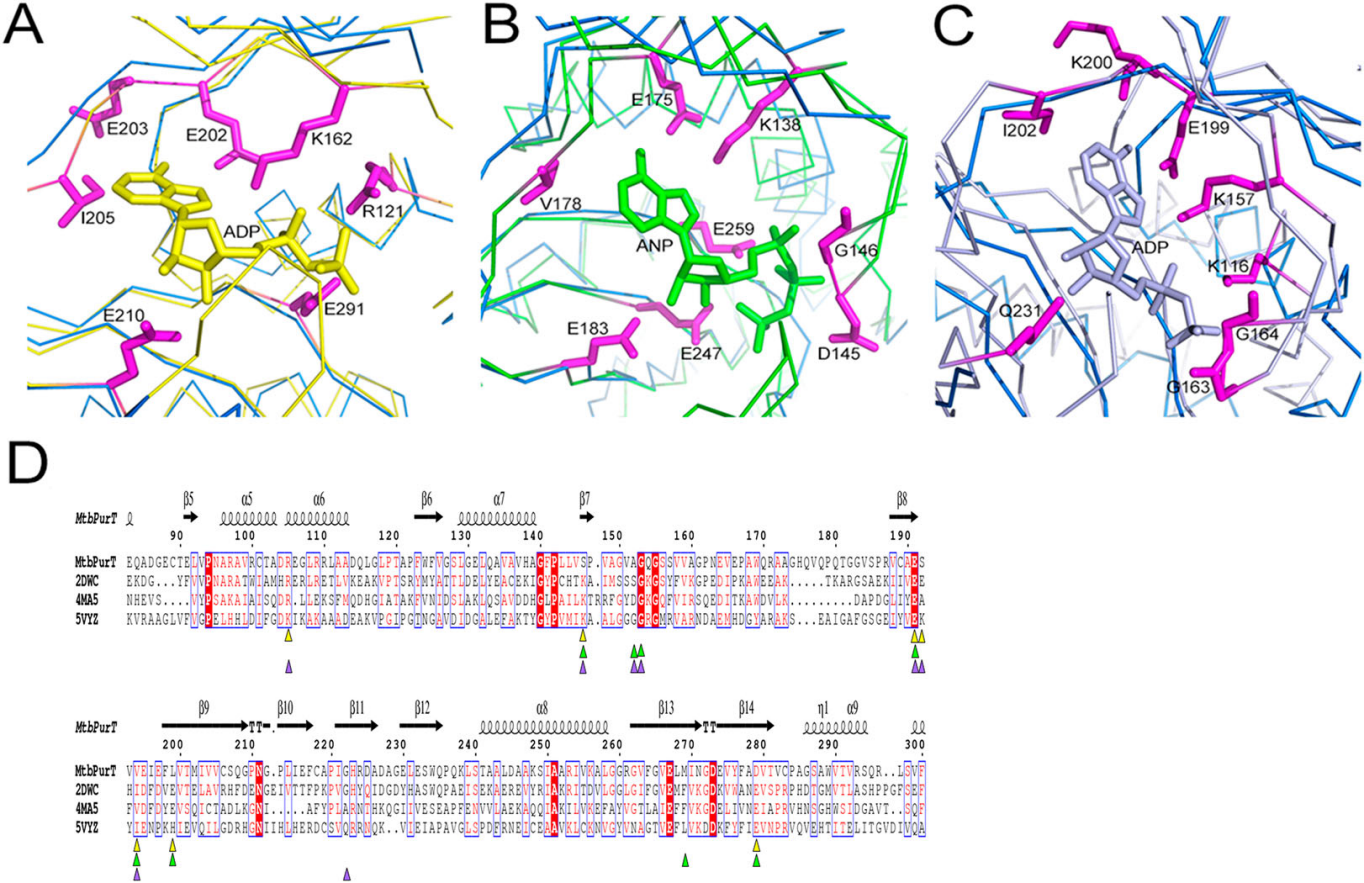
**Structural comparison of *Mtb*PurT with the ATP-grasp fold proteins.** Structure of *Mtb*PurT (coloured blue) was superposed with (A) *phBCCPP*urT-ADP (coloured yellow, PDB code: 2DWC, r.m.s.d: 1.31 Å), (B) *Ft*PurK-AMPPNP (coloured green, PDB code: 4MA5, r.m.s.d: 1.80 Å) and (C) *Ll*PC-ADP (coloured light purple, PDB code: 5VYZ, r.m.s.d: 2.44 Å). In each ATP-grasp fold protein, residues involved in cofactor binding are shown in sticks and coloured yellow, green and light purple, respectively. The corresponding residues in *Mtb*PurT are presented in sticks and coloured in magenta. (D) Multiple sequence alignment of *Mtb*PurT, *phBCCPP*urT, *Ft*PurK and *Ll*PC. Residues involved in binding with ATP-analogs are marked as triangles.

We compared the crystal structure of *Mtb*PurT with other known three-dimensional protein structures in the Protein Data Bank using DALI Server [33]. The top three hits are probable phosphoribosylglycinamide formyl transferase from *Pyrococcus horikoshii OT3* complexed with ADP (*phBCCPP*urT-ADP; PDB code: 2DWC; Dali Z-score: 42.0), *Francisella tularensis* phosphoribosylaminoimidazole carboxylase ATPase subunit complexed with AMPPNP (*Ft*PurK-AMPPNP; PDB code: 4MA5; Z-score: 33.7) and *Lactococcus lactis* pyruvate carboxylase complexed with ADP (*Ll*PC-ADP; PDB code: 5VYZ; Z-score: 27.7). The folding topology of *Mtb*PurT is remarkably similar to that of the members of the ATP-grasp fold proteins [34–37]. The structural superposition of *Mtb*PurT/*phBCCPP*urT-ADP (r.m.s.d: 1.31 Å, Figure 4(A)), *Mtb*PurT/*Ft*PurK-AMPPNP (r.m.s.d: 1.80 Å, Figure 4(B)) and *Mtb*PurT/*Ll*PC-ADP (r.m.s.d: 2.44 Å, Figure 4(C)) reveals that the ATP-binding motif of these proteins aligns well at the C-terminal and belongs to the ATP-grasp superfamily. The superimposition of these structures with *Mtb*PurT revealed that the amino acid residues for ATP binding share a surprising similarity. The corresponding residues contributing to this motif in *phBCCPP*urT-ADP are Arg121, Lys162, Glu202, Glu203, Ile205, Glu210 and Glu291 (Figure 4(A)); those in *Ft*PurK-AMPPNP are Lys138, Asp145, Gly146, Glu175, Val178, Glu183, Glu247 and Glu259 (Figure 4(B)); and those in *Ll*PC-ADP are Lys116, Lys157, Gly163, Gly164, Glu199, Lys200, Ile202 and Gln231 (Figure 4(C)). According to the sequence alignment, most residues are conserved for the binding with ATP-analogs (Figure 4(D)).

### A model of MtbPurT in complex with the cofactor ATP and substrate GAR

**Figure 5.**
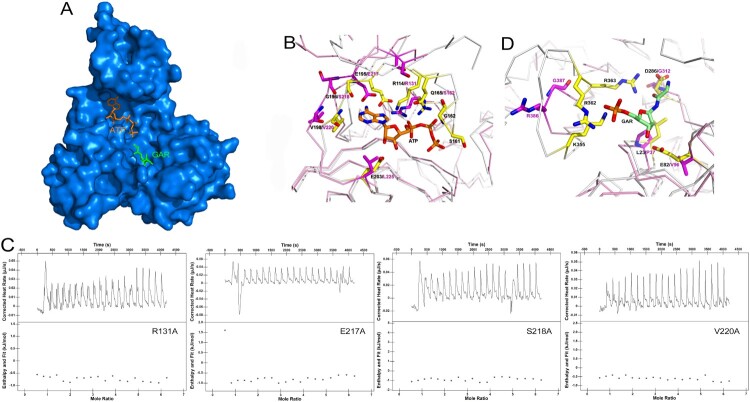
**Model of ATP and GAR binding to *Mtb*PurT.** (A) Surface representation of *Mtb*PurT with its proposed ATP and GAR. The ATP and GAR molecules are shown in stick representation. (B) The proposed models with ATP are displayed. Residues involved in ATP binding in *Ec*PurT are coloured yellow, and their corresponding residues in *Mtb*PurT are coloured magenta. (C) The ATP-binding affinity for *Mtb*PurT mutants was measured by ITC. None of the mutants bound ATPγS. (D) The proposed models with GAR are displayed. Residues involved in GAR binding in *Ec*PurT are coloured yellow, and their corresponding residues in *Mtb*PurT are coloured magenta. All residues are shown in stick representations.

It has been previously shown that the active site of PurT could be divided into two subsites, referred to the ATP-binding site and the GAR-binding site [38]. The ATP-binding site is positioned in the C terminus, while the GAR-binding site is located in the N terminus. To analyze the active binding pocket of *Mtb*PurT, we generated a model of *Mtb*PurT/ATP/GAR based on the superposition of the *Ec*PurT/ATP/GAR (PDB code: 1KJ8, r.m.s.d: 1.01 Å, Figure 5(A)). The model shows that the ATP molecule is primarily located on the pocket surrounded by the A- and C-domains. The amino acid residues surrounding the ATP-binding site share a remarkable degree of structural similarity to those of *Ec*PurT (Figure 5(B)). In the *Mtb*PurT/ATP/GAR model, the backbone carbonyl oxygen of Ser218 and the peptidic NH group of Val220 could contact the adenine ring of ATP, and these two amino acids are highly conserved in members of the ATP-grasp fold family. In addition, residue Glu217 also interacts with the adenine ring of ATP, which is consistent with Glu195 in *Ec*PurT. Residue Arg131 could generate a hydrogen bond to the phosphate group of ATP. Most of the residues for the ATP-binding in *Mtb*PurT are highly conserved as the *Ec*PurT structure. To validate our structural findings, we constructed four mutants of *Mtb*PurT, each carrying an amino acid substitution in the ATP-binding pocket. The binding affinity was also tested with ATPγS by ITC. The results were shown in Figure 5(C). All four mutants (R131A, E217A, S218A and V220A) lost their ATP-binding capability, indicating essential roles for these residues in binding with ATP. However, a long loop 175–180 between *β*7 and *β*8 involved in the interaction with the phosphate group of ATP is disorder, which needs to be investigated further.

In *Ec*PurT, the side chain of Glu82 forms hydrogen bonds with the hydroxyl groups of the GAR ribose, and the carboxylate side chain of Asp286 interacts with the amino group of GAR. Based on the sequence alignment, their corresponding residues Val96 and Gly312 in *Mtb*PurT have short side chains that no longer form hydrogen bonds with the GAR ribose. In *Ec*PurT, the side chains of residues Arg362 and Lys355 interact with the phosphoryl group of the GAR, and the guanidinium group of Arg363 contacts the carbonyl oxygen of GAR. However, the corresponding residues Arg386 and Gly387 in *Mtb*PurT show no hydrogen bonds with GAR. With their active sites superposed, the residues Arg362, Arg363 and Lys355 in *Ec*PurT were positioned in a long loop with 13 amino acids (residue 352–364), while Arg386 and Gly387 in *Mtb*PurT were located in a short loop with 7 amino acids (residue 383–389) (Figure 5(D)). A distance of 5.7 Å is observed between these two loops and the short loop in *Mtb*PurT is shifted away from the phosphoryl group of the GAR. Therefore, the structural differences suggested that the GAR binding would result in a large conformational change in *Mtb*PurT, and the catalytic mechanism of GAR should be different from that of *Ec*PurT.

## Discussion

Structural comparisons of *Mtb*PurT using DALI Server indicated that it was structurally similar to the ATP-grasp fold proteins with their characteristic three domains, and thus *Mtb*PurT is categorized as a member of the ATP-grasp superfamily as in the previous studies [13,39]. Each *Mtb*PurT monomer contains three structures referred to as the A-, B- and C-domains. Structural analysis of *Mtb*PurT indicated that the mycobacterial enzyme closely resembles the other bacterial PurT enzymes in terms of both overall fold and active site structure [19,32]. One of the most interesting results from a close inspection of the superimposed structure of apo-*Mtb*PurT over apo-*phBCCP*PurT structures is the difference in one loop around the phosphate groups of ATP. The loop 252–255 in apo-*Mtb*PurT adopts a position that defines an “open” conformation of the active site in the absence of ATP, in contrast with the “closed” conformation in *phBCCP*PurT. This rearrangement of residues and formation of the “open” conformation presents a broader binding pocket in the active site, although *Mtb*PurT exhibited similar ATP-binding affinity compared with *phBCCP*PurT. This noticeable difference in the ATP-binding pocket between *Mtb* and *phBCCP* PurTs was reflected in the biochemical structure-activity relationship studies and would provide crucial information for the design of more specific inhibitors.

Since PurT was found to catalyze the formylation of GAR using a catalytic mechanism requiring ATP and formate, it was of interest to understand how ATP and GAR bind to the protein. Therefore, we modelled both ATP and GAR into the *Mtb*PurT structure to seek an explanation for their binding mode. Model of *Mtb*PurT/ATP/GAR complex was generated based on the superimposition of *Ec*PurT/ATP/GAR. In the *Mtb*PurT/ATP/GAR model, the putative ATP-binding pocket in *Mtb*PurT was almost identical to other PurTs and the residues forming the ATP-binding pocket were well conserved among the PurTs. These residues such as Ser218, Val200, Glu217 and Arg131, form a hydrophobic pocket and play an important role in interacting with the ATP. In addition, the model of *Mtb*PurT/ATP/GAR shows that GAR is located on the pocket surrounded by the A- and C-domains. With the GAR-binding sites superposed, the residues of *Mtb*PurT surrounding the GAR-binding pocket showed a distinct difference from those of *Ec*PurT. In particular, loop 383–389 in *Mtb*PurT is much shorter than that in *Ec*PurT (loop 352–364) and shifted 5.7 Å away from the phosphate of the GAR substrate; thus, no hydrogen bonds with GAR were found. In fact, we performed docking calculations using AUTODOCK 4.2 [40] and tried to model the GAR into *Mtb*PurT. However, no output files were produced. Therefore, subtle differences in the loops that mediate substrate binding would markedly alter the *Mtb*PurT conformation and change the GAR binding site shape and contacts. The loop closure is a critical requirement for the binding of the GAR substrate and the subsequent catalysis.

In conclusion, the molecular structure of *Mtb*PurT has now been resolved at 2.79 Å resolution. PurT is found only in some eubacteria, including *Mtb*. Therefore, compounds that target *Mtb*PurT are attractive drug development options. This wealth of information might play an important knowledge base for the design of potential anti-TB agents in further. The inhibitors of *Mtb*PurT could contribute a possible opportunity and serve as useful lead compounds for anti-TB drug development.
